# Reproductive Tract Disorders among Afghan Refugee Women Attending Health Clinics in Haripur, Pakistan

**DOI:** 10.3329/jhpn.v28i5.6159

**Published:** 2010-10

**Authors:** Z.P. Balsara, I. Wu, D.R. Marsh, A.T. Ihsan, R. Nazir, E. Owoso, C. Robinson, G.L. Darmstadt

**Affiliations:** ^1^ Department of International Health, Bloomberg School of Public Health, Johns Hopkins University, Baltimore, MD, USA; ^2^ Save the Children-USA, Westport, CT, USA; ^3^ Save the Children-USA, Islamabad, Pakistan

**Keywords:** Maternal health services, Maternal welfare, Morbidity, Pelvic inflammatory disease, Prolapse, Afghanistan

## Abstract

Afghans comprise one of the largest groups of refugees in the world, with the majority living in Pakistan. The objective of this study was to identify commonly-occurring reproductive tract infections (RTIs), describe knowledge of women about RTIs, and assess physical and behavioural factors contributing to the development of RTIs. Afghan women presenting at Basic Health Units in refugee camps in Haripur, Pakistan, with reproductive health-related complaints, were included in the study (n=634). Data collection included implementation of an interviewer-administered questionnaire, along with a physical examination and laboratory tests. A descriptive analysis was conducted first. Qualitative data were coded and analyzed using predetermined themes. Chi-square test was used for determining the possible relationships between a binary outcome and categorical risk factors. Over three-fourths (76.7%) of those who reported to the health clinics with reproductive complaints had an RTI. Nearly half (49.5%) of these women were diagnosed with some form of vaginitis, and 14.7% were diagnosed with clinical suspicion of pelvic inflammatory disease (PID). Women with cervical prolapse (p=0.033) or who cleansed after intercourse (p=0.002) were more likely to have vaginitis. There was a significant difference (p=0.017) in the prevalence of suspected PID among women who used mud only (11.1%), any water (18.8%), and an old cloth or toilet paper (9.8%) for cleansing after defaecation. Specific physical and behavioural contributors to the high prevalence of RTIs in this population were identified, and recommendations to ameliorate these factors are offered.

## INTRODUCTION

Decades of strife have caused millions of Afghans to flee violence, political oppression, and economic instability (1) to become one of the largest groups of refugees in the world (2). Many live in neighbouring countries, such as Pakistan (2). Within Pakistan, an estimated 62% of Afghan refugees live in the Northwest Frontier Province (3).

In 1991, the Pakistan/Afghanistan office of Save the Children-USA joined the Safe Motherhood Initiative to provide healthcare to the refugees living in the Haripur camps in the Northwest Frontier Province. In 1998, as not much was known about the prevalence of morbidities due to RTIs within the Afghan refugee community, a clinical study was initiated to explore the extent of morbidity and possible associated behavioural factors. Much of past research on health of the refugee women has focused on women's delivery-related health, with particular emphasis on maternal mortality ratio (MMR). However, the MMR does not ade-quately represent other issues that can severely affect women's health, such as reproductive tract infections (RTIs). Untreated or improperly-treated RTIs can have severe, long-term sequelae, such as pelvic inflammatory disease (PID), infertility, ec- topic pregnancy, low birthweight of offspring, or foetal loss (4–6), and can increase a woman's and her community's risk of acquisition and transmission of HIV (7–9).

Since this study was conducted in 1998, there have been several political changes in the area with dramatic repercussions for Afghan refugees. Significant repatriation efforts are being exerted, resulting in an often-fluctuating refugee population (3). A recent report by the United Nations High Commissioner for Refugees (UNHCR) estimated that, as of end-2008, there were more than 2.8 million Afghan refugees. Approximately 1.8 million of these refugees continue to live in the UNHCR-supported refugee camps in Pakistan, with very little improvements in living conditions (2). Although this study was conducted in 1998, there have been no studies published in the intermediate period addressing morbidity due to RTIs among Afghan refugee women. Therefore, our findings remain highly relevant and urgent.

The objectives of this study were to assess the prevalence of RTIs and other gynaecological disorders among Afghan refugee women attending Basic Health Units (BHUs) in Haripur, Pakistan, and to describe the most commonly-occurring RTIs. Behavioural factors that might be contributing to the development of RTIs were also explored.

## MATERIALS AND METHODS

In 1998, there were 115,000 Afghan refugees of diverse ethnic groups, originating from different parts of Afghanistan, living in 18 refugee villages in Haripur. These refugee villages were served by a network of seven BHUs. The catchment area in this study included all the seven BHUs. In 1996, a verification exercise estimated that there were about 22,540 (19.6%) women of reproductive age among the Hairpur refugee population.

### Training and pre-study preparations

Each BHU was staffed by a primary healthcare (PHC) team, headed by an Afghan female physician and a rotating male doctor catering to the gender-specific needs of the male population. Fieldwork was conducted from January to mid-March 1999. Before commencing fieldwork, a five-week preparatory phase of the project began in November 1998. During this phase, it was ensured that all the seven BHUs had the necessary equipment and materials required for the study. Medical Officers received training on the syndromic guidelines of the World Health Organization, diagnosis and management of reproductive tract disorders and other clinical issues, such as vaginal ecosystem, normal and abnormal flora, factors affecting vaginal flora, etc. Medical Officers also received training on interviewing techniques, collection of vaginal swab, plating of specimens, and preparation of slides. Field-testing of the questionnaire was conducted in the latter part of December 1998.

### Study population

Trained female study physicians attended the refugee women who presented at the BHUs with reproductive health complaints. All women who came in with a reproductive health complaint from January to mid-March 1999 were asked to participate in the study. Women on antibiotic treatment in the preceding three weeks were excluded from the study to limit the impact of antibiotic regimens on laboratory results obtained for other aspects of the study. Women who agreed to participate gave verbal informed consent and were assured of confidentiality and anonymity. Those who refused to participate were treated without prejudice. No further data were collected on women excluded from the study. The number of women excluded for the previous reasons was not recorded.

### Data collection

An interviewer-administered questionnaire was used for delving into each participant's medical, social and behavioural history relating to reproductive health-related morbidity. The questionnaire was administered by a female physician who was fluent in Pashto, the most widely-spoken language in the refugee villages. Using a blend of open- and close-ended formats, the questionnaire enabled the physician to check all self-reported symptoms in a list and probe further into each of the complaints that were raised by the participants. Questions were also asked to determine marital and obstetric history, past and present therapeutic interventions, and contraceptive-use. The questionnaire also explored specific behaviours, such as personal hygiene practices during menstruation, after intercourse, and after defaecation.

After completing the interview, the patient had a thorough examination which included a general, abdominal and pelvic (i.e. speculum and bimanual) examination. The purpose of this examination was to detail any abnormality, particularly in the reproductive tract or related organ-systems. Since comprehensive vaginal examination was not routinely done in the past, the physicians explained thoroughly the rationale for the procedure and also used the opportunity to ask sensitive questions to which reliable responses could not be expected in the ‘face-to-face’ interview. A wet prep and potassium hydroxide (KOH) test of vaginal discharge was done on-site if there was suspicion of vaginitis, and treatment was provided.

The pelvic examination culminated in the collection of swabs and specimens for laboratory analysis. The specimens were transported daily from the BHUs to Islamabad, a journey lasting about one and a half hours.

The definitions of reproductive health-related morbidities and provisional diagnoses used in this study are outlined in Table 1.

**Table 1.** Definitions of reproductive tract disorders and diagnoses in the study

Disorder	Diagnostic criteria
Cervical prolapse (10)	
1^st^ degree	Bulging of vaginal walls and/or descent of the uterus where the cervix is >1 cm above the hymen
2^nd^ degree	Bulging of vaginal walls and descent of the uterus with the cervix showing at the vulva, with the cervix <1 cm above or below the hymen
3^rd^ degree	Most distal portion of the prolapse is no greater than 2 cm below the hymen
Cystocele	Anterior wall laxity
Rectocele	Posterior wall laxity
Vaginocele	Hernia into the vagina
Suspicion of pelvic inflammatory disease	Constant lower abdominal pain and cervical excitation tenderness
Cervicitis	Redness and/or oedema of the cervix on speculum examination
Vaginitis *Trichomonas*	Abnormal discharge, physical discomfort, and trichomonads on wet prep examination
Candidiasis	Abnormal discharge, physical discomfort, vaginal redness, and hyphae on wet prep examination
Bacterial vaginosis	Abnormal discharge, physical discomfort, positive Whiff test, and detection of clue cells on laboratory examination
Infertility	Inability of a sexually-active woman not on contraceptives to achieve conception or to carry a pregnancy to livebirth despite consistent attempts over >1 year

### Analysis of data

Data were analyzed using the Stata® software (version 9.0) (Stata Corp, College Station, TX, USA). A descriptive analysis of spontaneously-reported reproductive health-related morbidities and symptoms, prompted reporting of morbidities, and reporting of behavioural factors relating to hygiene during menstruation, after defaecation, and after intercourse was conducted. Furthermore, using a chi-square test with statistical significance defined as p<0.05, analysis of data focused on measuring the impact of certain behavioural factors, such as cleansing after intercourse, cleansing after defaecation, and method used for absorbing menses on the prevalence of RTIs.

Qualitative data were coded based on predetermined themes, which included behaviours relating to cleansing after defaecation, during menstruation, after sexual intercourse, barriers to healthcare-seeking, and perceived possible causes of RTIs. Responses to qualitative inquiries were used for supplementing data on behaviours reported through the structured questionnaire.

## RESULTS

### Population demographics

In total, 634 women who attended the seven BHUs during the study period with a reproductive health problem were eligible for participation and provided verbal informed consent. The average age was 29.6 years [standard deviation (SD) 7.8], and the median age was 30 (range 12-70) years. Most (98.6%) women were married, 22.8% had no children, 29.2% had 1-3 children, 40.7% had 4-9 children, and 7.0% had 10 or more children.

### Reported symptoms and healthcare-seeking behaviours

*Unprompted responses*: The most commonly-reported symptoms are presented in Table 2. In general, lower proportions of the women reported symptoms unprompted (open-ended questions of the questionnaire) compared to when prompted (structured section of the questionnaire). The most common unprompted reproductive health concerns reported were lower backache (78.2%), ‘a feeling of wetness below’—indicating vaginal discharge (76.5%), and lower abdominal pain (72.7%).

**Table 2.** Symptoms of Afghan refugee women (n=634) reporting to Basic Health Units with reproductive health complaints (more than one symptom was possible)

Response	No.	%
Self-reported symptoms	
Vaginal discharge	485	76.5
Genital pruritus	193	30.4
Dyspareunia	246	38.8
Lower abdominal pain	461	72.7
Infertility	166	26.2
Prolapse	104	16.4
Lower backache	496	78.2
Prompted response		
Vaginal discharge	571	90.1
Excessive	312	54.6
Foul smelling	145	25.4
Abnormal colour	216	37.8
Lower abdominal pain	575	90.7
During intercourse	253	44.0
During menses	299	52.0
Constant	209	36.3
Dyspareunia	442	69.7
Prolapse	383	60.4

*Prompted responses*: When prompted, 69.7% of the women reported having pain during sexual intercourse (dyspareunia). Approximately one in four women [27.0% (119/442)] reporting symptoms of dyspareunia had previously sought treatment from a physician. One woman tried a home remedy: ‘mixture of milk and raw egg, daily for one week’. Over 70% (n=322) of the 442 women reported not seeking any care. Of them, 216 mentioned a specific reason for not seeking medical help. Of the 216 women, 27.3% (n=59) cited shame, 33.8% (n=73) mentioned lack of finances, and 18.5% (n=40) mentioned lack of familial support as the primary reason for not seeking help. Of this latter group, many women categorically stated: “my mother-in-law did not allow me to seek help.” One woman said, “my husband is a mullah”—implying the inherent difficulty in discussing such an intimate and sensitive issue with her religious leader husband. Seventeen (7.9%) of the 216 women believed that pain upon intercourse was natural or were unaware that treatment was available.

Three hundred eighty-three (60.4%) of the 634 women answered ‘yes’ to the question: “Do you feel heaviness below—as if something is falling out from under you.” One hundred eighty-three (47.8%) of 383 women sought help for this problem from a physician. Of 168 women who had not sought any medical help for this symptom, the most common reasons mentioned were shame (13.1%), lack of financial means (34.5%) and lack of cooperation from their husbands and/or mothers-in-law (29.8%). Of 337 women who reported possible causes of prolapse, 53.1% attributed it to pregnancy-related causes but other causes mentioned included the use of contraceptives, caesarian sections, cough, and having a fall (23.2%). However, 23.7% could not provide an explanation or cite a cause of the prolapse.

One hundred sixty-six (26.1%) of the 634 respondents had problems conceiving a child or carrying a pregnancy to term. One hundred ten (66.3%) of this group (n=166) were primarily infertile, i.e. they had no living child. The remaining one-third of the women with infertility had secondary infertility but had been unable to conceive for at least the past year.

### Reported behaviours

The most common method of cleansing after defaecation was the use of mud. Of the 634 women, 61.8% ever-used this method, and 38.3% used this method exclusively. On prompting, it was revealed that dried mud was perceived to be a clean substance. Of the 634 women, 48.7% sometimes used water for cleansing after defaecation, 8.7% used an old cloth, and 20% used toilet paper.

Of the 634 women, 81.2% cleaned their vaginal area after intercourse with an old cloth, 4.1% douched with water and soap, and 1.4% used toilet paper. Most (92%) women used a washed old cloth or rag to absorb menstrual flow, 1% used an unwashed old cloth, and 5% reported not using anything at all.

### Clinical signs

On clinical examination, 50.5% of the 634 women had copious amounts of discharge, and the majority (61.5%) of the women reporting any discharge described it as ‘white and curdy’ (Table 3). Although 28.2% of the women had discharge associated with an offensive odour, only 6.6% had a positive KOH test. Three hundred forty-two (53.9%) of the 634 women were diagnosed with cervical prolapse on pelvic examination. Degrees of prolapse are presented in Table 3.

**Table 3.** Physical findings on clinical examination of Afghan refugee women (n=634) reporting with reproductive health complaints

Physical sign	No.	%	Physical sign	No.	%
Lower abdomen			Cervix		
Tender	483	76.2	Eroded	300	47.3
Vaginal wall			Oedema	338	53.3
Inflamed	401	63.2	Inflammed	259	40.9
Suppurative	20	3.2	Lacerated	69	10.9
Ulcerations	13	2.1	Friable	200	31.5
Fistula	0	0.0			
Origin of discharge			Bimanual examination		
Vaginal	201	31.7	Cervical excitation tenderness	323	50.9
Cervical	76	12.0	Uterine tenderness	383	60.4
Vaginal/cervical	390	61.5	Right adnexal tenderness	383	60.4
Urethral	2	0.3	Left adnexal tenderness	343	54.1
Discharge			Cervical prolapse	342	53.9
Excessive	320	50.5	1^st^ degree	91	14.4
White and curdy	390	61.5	2^nd^ degree	96	15.1
Mucopurulent	88	13.9	3^rd^ degree	10	1.6
Green	17	2.7	Cystocele	269	42.4
Offensive odour	179	28.2	Vaginocele	78	12.3
Positive KOH test	42	6.6	Rectocele	160	25.2

KOH=Potassium hydroxide

### Diagnoses and possible risk factors

The most common provisional diagnosis was vaginitis (Table 4). One hundred forty-nine (50.0%) of the 634 women were diagnosed with trichomoniasis, bacterial vaginosis, or candidiasis. Two hundred fifty (39.4%) of the 634 women were diagnosed with cervicitis, and 14.7% were diagnosed with suspicion of clinical PID. The overall rate of upper or lower RTI was 76.7%. Table 1 describes the definition used for each of these diagnoses.

**Table 4.** Rates of and risk factors for reproductive tract infections diagnosed among Afghan refugee women (n=634) reporting with reproductive health complaints

Physical finding or behaviour	Diagnosis				
	Vaginitis (n=314, 49.5%)	Suspicion of clinical PID (n=93, 14.7%)	Cervicitis (n=250, 39.4%)	Gonorrhoea/ chlamydia (n=105, 16.6%)	Lower or upper RTI (n=486, 76.7%)
Prolapse					
None	45.6	20.2	41.2	14.3	80.4
Any	54.1	8.2	37.3	16.8	72.3
p value	0.033	<0.001	0.317	0.394	0.016
Method to absorb menses					
None	39.4	33.3	42.4	6.06	69.7
Washed cloth	49.8	13.3	39.2	16.2	46.8
Other	60.0	26.7	40.0	6.61	86.7
p value	0.361	0.003	0.935	0.185	0.419
Cleansing after intercourse					
None	28.9	15.4	32.7	7.7	55.8
Yes	51.4	14.6	40.0	16.1	78.5
p value	0.002	0.879	0.229	0.106	<.001
Cleansing after toilet					
Mud only	54.3	11.1	41.6	15.6	78.2
Any water	45.0	18.8	35.3	15.3	74.1
Other	52.4	9.8	51.2	14.6	81.7
p value	0.080	0.017	0.058	0.975	0.271

PID=Pelvic inflammatory disease;

RTI=Reproductive tract infection

Over half (54.1%) of the women who were diagnosed as having prolapse were also diagnosed as having vaginitis. The women who had prolapse were significantly more likely to also have vaginitis (p=0.033) compared to the women who did not have prolapse. There was a significant difference in the prevalence of suspected PID among the women who used a washed cloth to absorb menses (13.3%), women who used nothing (33.3%), and women who used other methods (26.7%), such as unwashed cloth (p=0.003).

The women who cleansed after intercourse (51.4%) were significantly more likely to be diagnosed as having vaginitis (p=0.002) compared to the women who did not cleanse after intercourse. There was a significant difference (p=0.017) in the prevalence of suspected PID among the women who used mud only (11.1%), any water (18.8%), and an old cloth or toilet paper (9.8%) for cleansing after defaecation.

## DISCUSSION

Of the 634 women who visited the BHUs with reproductive health complaints during the study period, 76.7% were given a provisional diagnosis of an RTI. The results of the study indicated that women who had prolapse were more likely to have vaginitis. Cervical prolapse compromises the integrity of the vaginal wall and interrupts the delicate equilibrium of the vaginal ecosystem, increasing the potential for the development of infection (11). Over half of the study women had some degree of genital prolapse. There have been few studies on the prevalence of prolapse among women in low-resource settings, with rates ranging from 31.7% in Korea (12) to 46% in rural Western Gambia (13) and 53% in Iran (14). The prevalence rate (54%) in our study is similar to those in the last two studies, likely because the demographic characteristics and the socioeconomic status of those women are more similar to those of our study cohort. Additionally, the relative frequency of the different stages in our cohort mirrored those observed in other studies, where the majority of women with prolapse had stage 1 or stage 2 disease (12,14). However, ours was a facility-based study of women who presented themselves at the seven BHUs, thus, limiting generalization to the whole population of Afghan refugees in the Haripur area.

A study by the United Nations Children's Fund reported that 54% of women in Afghanistan are married by the age of 18 years (15). The average age-at-marriage was approximately 15 years in rural areas (16) and 17 years in urban areas (17). Women are expected to bear children immediately after marriage, often do not use contraception (18) and bear, on average, 7.5 children throughout their reproductive life (1). These Afghan cultural norms for multiparity, beginning immediately after early marriage, likely contribute to these women having high rates of prolapse, which is a reproductive health-related morbidity and risk factor for the development of RTIs.

Personal hygiene behaviours of women may play a role in increasing their risk for developing an RTI. The use of mud or water to cleanse after defaecation was associated with suspicion of clinical PID. With the physical proximity of the anus to the female genitalia, it is possible that mud may enter and contaminate the vagina. Additionally, the water that the respondents use for washing poses another source of contamination. Pipe-borne water is not available in the refugee villages, and most areas are served by boreholes. These boreholes frequently become contaminated, particularly during the rainy season when the nearby lake overflows. Cleansing after intercourse may also contribute to the development of RTIs (19). Nearly 80% (78.5%) of the women who cleansed after intercourse had a diagnosis of RTI compared to 55.8% of the women who did not. Cleansing after intercourse was also associated with the diagnosis of vaginitis. The most common methods used for cleansing after intercourse in this population were the use of an old cloth (81%) and douching with soap/water (4%). Douching after intercourse has been identified as a risk factor for RTIs by changing vaginal pH and suppressing endogenous bacteria (20). Similar unhygienic practices during menses may introduce pathogens into the reproductive tract, with previous studies documenting a direct association between poor menstrual hygiene and RTIs (5,21). Our data showed that the use of a washed cloth to absorb menses was associated with a lower risk of suspicion of clinical PID.

Many Afghan refugee women may not be aware of potential warning-signs of RTIs. Dyspareunia may be a sign of several reproductive infections (22) but more than half of the study women with dyspareunia did not seek treatment. Some women simply believed that pain upon sexual intercourse was normal whereas others used home remedies. Results of other studies have similarly demonstrated a lack of knowledge on reproductive health among Afghan women (17). Clearly, there is a role for reproductive health education. Over one-third of the women with dyspareunia or prolpase reported that they did not have the financial resources to seek treatment. Other women cited lack of permission from either their husbands or mothers-in-law for not seeking care. Another study on Afghan women in Pakistani refugee camps found that the majority of married women lived with the family of their husbands, where the parents-in-law are often the decision-makers (23).

Untreated or improperly-treated RTIs may lead to severe consequences, including infertility. One quarter of the study women had problems with conception or bearing a child to term. In Afghan society, a woman's fertility and ability to reproduce is of high importance (17). Our study did not address intimate partner violence (IPV) but several studies have demonstrated an association between physical and verbal IPV and the prevalence of RTIs (24–26). Seventy-nine percent of Afghan women in refugee camps in Pakistan had been physically abused by their husbands (27). Given the widespread acceptance of a certain level of IPV (23) and the high rates of infertility in our study cohort, a further study should be conducted to explore the role of IPV in the development of RTIs in this population and the role of RTIs in the incidence of IPV.

### Limitations

There are likely other causes for RTI in this population of vulnerable women who have not been explored in this study. There was selection bias in the enrollment process as those interviewed were women who self-reported to the BHUs for reproductive health complaints. Thus, the results of this study, particularly morbidity prevalence rates, cannot be extrapolated to the Afghan refugee community as a whole. These women could be relatively more proactive about their health. However, the study also excluded those women who had been on antibiotics. These women may have had more financial resources to seek care. Nevertheless, this study was intended to add to our understanding of the types of complaints, and the behavioural and physical factors that may lead to the development of RTIs. Since this study does not have a population-based control group for use as a comparison, the associations between behaviour and symptoms may be taken as indicative but not definitive in this population.

### Conclusions

Despite limitations, this study highlighted the burden of reproductive health problems and identified possible behavioural risk factors for further study.

Since the study was conducted in 1999, there have been dramatic political changes in the area. During 2001-2006, many camps in Pakistan were closed as 2.4 million Afghan refugees returned to their home country (1). In a recent census of Afghans in Pakistan, however, roughly 83% reported that they had no plans to return to Afghanistan, and approximately 2.8 million Afghans remain in Pakistan (2,3). Those Afghans who do return to Afghanistan continue to face health challenges. Although the Taliban rule was overthrown in 2001 and a new democratic regime installed, this change has not significantly improved women's status or healthcare (28–30). The MMR remains among the highest in the world (30–32).

This study revealed the high prevalence of RTIs among women attending the seven BHUs for reproductive health concerns. Additionally, several behavioural and social factors that could possibly be risk factors for the development of RTIs and the possible barriers to seeking care for RTIs were identified. Further studies should be conducted to better understand these relationships. Based on our findings, there is a need to develop informational and educational programmes and packages that inform women about the signs, symptoms, and sequelae of RTIs and other reproductive health-related morbidities. Educational messages should target both, women returning to Afghanistan from Pakistan, and those remaining in the refugee camps in Pakistan.

## ACKNOWLEDGEMENTS

The authors thank Dr. Zeba Rasmussen for her helpful comments on an earlier draft of the project report.
